# Clinical outcomes and healthcare costs of inpatients with tetanus in Korea, 2011–2019

**DOI:** 10.1186/s12879-021-05935-w

**Published:** 2021-03-09

**Authors:** Sohyun Bae, Minsik Go, Yoonjung Kim, Soyoon Hwang, Shin-Woo Kim, Ki Tae Kwon, Sook-In Jung, Hyun-Ha Chang

**Affiliations:** 1 Division of Infectious Diseases, Department of Internal Medicine, School of Medicine, Kyungpook National University, 130 Dongdeok-ro, Jung-gu, Daegu, 41944 South Korea; 2 Department of Internal Medicine, Kyungpook National University Hospital, Daegu, South Korea; 3 Department of Infectious Diseases, Chonnam National University Medical School, 42 Jebongro, Donggu, Gwangju, 61469 South Korea

**Keywords:** Tetanus, Healthcare costs, Treatment outcome

## Abstract

**Background:**

Tetanus is a rare, vaccine-preventable but extremely serious disease. We investigated the recent trend of the clinical outcomes and medical costs for inpatients with tetanus in South Korea over 10 years.

**Methods:**

We conducted a retrospective review to determine the clinical factors and medical costs associated with tetanus at two national university hospitals in South Korea between January 2011 and October 2019.

**Results:**

Forty-nine patients were admitted for tetanus (mean age, 67.0 years [range, 53.0–80.0 years]; 32 women [57.1%]). All the patients had generalized tetanus, and 5 (10.2%) died during hospitalization. The median duration from symptom onset to hospital admission was 4 days. Trismus (85.7%) was the most common symptom, and the median hospital stay was 39 days. Thirty-two patients (65.3%) required mechanical ventilation, and 20 (40.8%) developed aspiration pneumonia. The median total healthcare cost per patient was US $18,011. After discharge, 35 patients (71.4%) recovered sufficiently to walk without disability.

**Conclusions:**

Tetanus requires long hospital stays and high medical expenditures in South Korea; however, the vaccination completion rate is low. Medical staff should therefore promote medical advice and policies on the management of tetanus to the general South Korean population.

## Background

Tetanus is a fatal but preventable infectious disease caused by *Clostridium tetani.* According to the US Centers for Disease Control and Prevention, tetanus vaccines have largely contributed to the reduced tetanus surveillance and morbidity since 1990 [1]. Similarly, the incidence of tetanus in South Korea has decreased since the introduction of the diphtheria and tetanus toxoid and acellular pertussis vaccine in 1982 [2].

As recommended by the Committee on Infectious Disease of the Korean Pediatric Society, the tetanus immunization schedule for children is five doses of diphtheria and tetanus toxoids and acellular pertussis vaccine at 2, 4, 6, 15, and 18 months of age, and then again at 4–6 years. This schedule should be followed by a tetanus toxoid, diphtheria, and acellular pertussis (Tdap) vaccine dose at 11–12 years of age, and a tetanus toxoid vaccine (Td) dose every 10 years [3]. However, insufficient tetanus vaccinations have been administered to adults in certain cases of tetanus. According to the Korea Centers for Disease Control and Prevention (KCDC), the nationwide incidence of tetanus increased to a mean of 23.9 cases per year between 2010 and 2019 as compared with the mean of 10.9 cases per year between 2000 and 2009. Given the steady increase in its incidence, tetanus remains difficult to prevent and treat. Unfortunately, studies are limited regarding tetanus cases in South Korea. The most recent study on the clinical manifestations of tetanus was conducted at Chonnam National University in 2003. It focused on the clinical and epidemiological findings of tetanus from 17 cases in South Korea, with no mention regarding the cost incurred for the treatment of the disease. We therefore aimed to investigate the clinical features and inpatient costs of tetanus within the past 10 years.

## Methods

### Data collection

This retrospective cohort study was conducted between January 1, 2011 and July 30, 2019 at Kyungpook National University Hospital and Chonnam National University Hospital. The definition of patients with tetanus was extracted from the nomenclature of the *International Classification of Disease, Tenth Revision* (*ICD-10*). Two physicians reviewed the patient data that included demographics, clinical characteristics, and laboratory findings retrieved from the hospital medical records and data. All the included patients were older than 18 years and diagnosed as having tetanus at 1 of 2 tertiary referral centers. We did not include neonatal tetanus. Tetanus was defined according to the clinical features, such as generalized or localized muscular spasm and contractures without other medical causes. As defined in this study, generalized tetanus is characterized by painful muscle spasms of the jaw and neck, muscular contraction unrelated to injury sites, or autonomic dysfunction, and patients who presented fluctuating systolic blood pressure or heart rate [4] or required inotropic agents or calcium channel blockers to control their blood pressure were defined as having autonomic dysfunction. The patients were divided into two groups, a non-mechanical ventilation (nonMV) group and a mechanical ventilation (MV) group. The MV group included patients who received invasive ventilation during hospitalization, while the nonMV group included patients who were not administered ventilation. We collected data on the patients’ demographic characteristics, tetanus-related clinical features, hospital stay duration, rehabilitation treatment duration, infection source, complications during treatment, and medical costs for tetanus treatment. Aspiration pneumonia was confirmed by a radiologist by performing chest radiography or CT scans, the results of which were reviewed by a physician. The requirement for rehabilitation was determined by consultation with the rehabilitation professionals. We considered loss of immunization visits when the patients did not attend the arranged outpatient clinic visit on time and no medical referral was indicated in the electronic medical records. We also collected data on the laboratory results and expenditures during the tetanus treatment. The medical charts from most of the patients were reviewed during their first and last hospital visits.

### Statistical analyses

All statistical data were analyzed using R statistics version 3.1. The categorical variables are expressed as numbers and percentages and were compared using the chi-square test or Fisher exact test. The continuous variables are expressed as mean ± standard deviation (SD) or median with the interquartile range (IQR) and were compared using the Mann–Whitney *U* test. Data that did not exhibit a normal distribution are represented as median with IQR. We used a multivariate logistic regression analysis to evaluate the risk factors associated with MV. *P* values < 0.05 were considered statistically significant.

### Ethics statement

The institutional review board of Kyungpook National University Hospital and Chonnam National University Hospital reviewed and approved our study protocol (approval Nos. KNUH-201907031 and CNUH-2019235, respectively). Considering the retrospective nature of the study and the use of anonymous clinical data for the analysis, the requirement for informed consent was waived.

## Results

### Patient characteristics and clinical features

We identified 49 patients aged ≥18 years who were hospitalized for tetanus, of whom 32 required invasive MV. Table 1 lists the baseline characteristics and clinical presentation of the MV and nonMV groups. Among the 49 patients with tetanus, 32 (57.1%) were women, and the median age was 67.0 years (IQR, 53.0–80.0). The demand for MV increased significantly among the older patients (median age, 72.5 years [IQR, 59.0–82.0 years] and 53.0 years [IQR, 46.0–67.0 years] for the MV and nonMV groups, respectively; *P =* 0.002). The incubation time was shorter for the nonMV group than for the MV group but did not differ significantly (median, 6.0 days [IQR, 3.0–8.0 days] vs. 2.0 [IQR, 1.0–6.0 days]; *P =* 0.237). All the patients developed generalized tetanus. With regard to the entry site of the toxin, an unknown entry site (34.7%) was the most common, followed by the legs (24.5%) and arms (22.4%). The most common symptoms on admission were trismus (85.7%) and muscle stiffness (73.5%). Most patients developed autonomic nervous dysfunction, and half of the patients (51.0%) developed fever during their hospital stay.

**Table 1:** Characteristics of the patients with tetanus according to the mechanical ventilation required

Factors	Total (*n* = 49)	No mechanical ventilation (*n* = 17)	Mechanical ventilation (*n* = 32)	*P* value
**General characteristics**				
Women, *n* (%)	32 (57.1%)	11 (64.7%)	21 (65.6%)	0.949
Median age, years [IQR]	67.0 [53.0; 80.0]	53.0 [46.0;67.0]	72.5 [59.0;82.0]	0.002
Median incubation time, days [IQR]	5.0 [1.5–7.5]	2.0 [1.0–6.0]	6.0 [3.0–8.0]	0.237
Median duration from symptom onset to admission, days [IQR]	4.0 [2.0–9.0]	4.0 [2.0–6.5]	7.0 [5.0–1.5]	0.120
Wound operation	6 (12.2%)	3 (18.8%)	3 (12.5%)	0.928
**Comorbidity,** ***n*** **(%)**				
Hypertension	16 (32.7%)	3 (17.6%)	13 (40.6%)	0.189
Diabetes mellitus	11 (22.4%)	3 (17.6%)	8 (25.0%)	0.820
Malignancy	8 (16.3%)	5 (29.4%)	3 (9.4%)	0.161
Others	17 (34.7%)	4 (23.5%)	13 (40.6%)	0.378
**Site of entry,** ***n*** **(%)**				0.314
Legs	12 (24.5%)	5 (29.4%)	7 (21.9%)	
Arms	11 (22.4%)	5 (29.4%)	6 (18.8%)	
Face	7 (14.3%)	7 (15.9%)	0	
Trunk	7 (14.3%)	0 (0.0%)	7 (21.9%)	
Cryptogenic	17 (34.7%)	6 (35.3%)	11 (34.4%)	
**Symptom during hospitalization,** ***n*** **(%)**				
Trismus	42 (85.7%)	14 (82.4%)	28 (87.5%)	0.951
Muscle stiffness	36 (73.5%)	7 (41.2%)	29 (90.6%)	0.001
Swallowing difficulty	33 (67.3%)	9 (52.9%)	24 (75.0%)	0.212
Dysarthria	32 (65.3%)	11 (64.7%)	21 (65.6%)	1.000
Opisthotonos	11 (22.4%)	2 (11.8%)	9 (28.1%)	0.344
Seizure	11 (22.4%)	2 (11.8%)	9 (28.1%)	0.344
Fever	25 (51.0%)	5 (29.4%)	20 (62.5%)	0.057
Nausea and vomiting	2 (4.1%)	1 (5.9%)	1 (3.1%)	1.000
Sialorrhea	2 (4.1%)	–	2 (6.2%)	0.769
Dyspnea	28 (57.1%)	5 (29.4%)	23 (71.9%)	0.011
Autonomic dysfunction	43 (87.8%)	11 (64.7%)	32 (100%)	0.002

*Abbreviation*: *N* number, *IQR* interquartile range

### Clinical findings of the patients who died

The median age of the five patients who died was 85 years (range, 77–89 years), and four (80%) of these patients were women. Four of the five patients had no definitive injuries and did not know the incubation time. In one case, the dorsum of the right hand was injured 14 days prior to symptom onset. Before admission, none of the five patients remembered their tetanus vaccination history. Four patients were provided with MV, and one patient refused life-sustaining treatment, including MV and cardiopulmonary resuscitation because of their advanced age. The cause of death in all five patients was respiratory failure. The tetanus complications in this group included aspiration pneumonia in two patients, pulmonary thromboembolism in one patient, and multifocal infarction in one patient.

### Clinical outcomes and significant factors of mechanical ventilation status

Table 2 provides the details of the clinical outcomes and complications. Twenty patients (40.8%) experienced aspiration pneumonia, and 18 (36.7%) had concurrent ventilator-associated pneumonia. Twenty-one (65.6%) cases of nosocomial infection were reported in the MV group, and three (17.7%) were reported in the nonMV group.

**Table 2:** Clinical outcomes of the patients with tetanus according to the mechanical ventilation required

Factors	Total (*n* = 49)	No mechanical ventilation (*n* = 17)	Mechanical ventilation (*n* = 32)	*P* value
**Complications,** ***n*** **(%)**				
Aspiration pneumonia	20 (40.8%)	2 (11.8%)	18 (56.2%)	0.007
Pulmonary thromboembolism	2 (4.1%)	0	2 (6.2%)	
Multifocal infarction	1 (2.0%)	0	1 (3.1%)	
Urinary tract infection	3 (6.1%)	0	3 (9.3%)	
Atrial fibrillation	1 (2.0%)	0	1 (3.1%)	
Other	8 (16.3%)	1 (5.9%)	7 (21.8%)	
**Clinical outcome**				
Median hospital stay, days [IQR]	39.0 [9.0;49.0]	8.0 [5.0–21.0]	46.5 [39.5–52.5]	< 0.001
Median intensive care unit stay, days [IQR]	24.0 [2.0;38.0]	0.0 [0.0;3.0]	33.5 [24.5;41.5]	< 0.001
Median duration of mechanical ventilation, days [IQR]	–	–	33.0 [22.0–40.5]	
Tracheostomy	27 (55.1%)	–	27 (84.4%)	
Ambulation at discharge	35 (71.4%)	13 (92.9%)	22 (81.5%)	0.609
Death	5 (10.2%)	1 (5.9%)	4 (12.5%)	0.816
Rehabilitation	15 (24.5%)	1 (5.9%)	11 (34.4%)	0.063
Second tetanus vaccination	24 (49.0%)	3 (17.6%)	21 (65.6%)	0.004
Third tetanus vaccination	6 (12.2%)	1 (6.2%)	5 (16.1%)	0.617

*Abbreviation*: *N* number, *IQR* interquartile range

Thirty-two (84.2%) of the 38 patients admitted to the intensive care unit (ICU) required MV. The median MV duration was 33.0 days (IQR, 22.0–40.5 days), and the hospital stay was longer for the MV group than for the nonMV group (median, 46.5 days [IQR, 39.5–52.5 days] vs. 8.0 days [IQR, 5.0–21.0 days]; *P* <  0.001). Eleven patients (34.4%) in the MV group required rehabilitation, whereas only one patient (5.9%) in the nonMV group required rehabilitation. The median rehabilitation period was 30.0 days (IQR, 13.0–39.0 days) for the MV group. One patient in the nonMV group underwent rehabilitation for 54.0 days. Most of the patients recovered without impaired motor function after discharge, and the degree of recovery demonstrated no significant difference between the groups (*P =* 0.609). The proportion of secondary tetanus vaccinations was significantly higher in the MV group (65.6%) than in the nonMV group (17.6%; *P =* 0.004), whereas the proportion of third tetanus vaccinations was not significantly different between the MV (16.1%) and nonMV groups (6.2%; *P =* 0.617).

As shown in Table 3, age > 65 years and the presence of dyspnea were identified as risk factors for the need of MV support in the multivariate regression logistic analysis (age 65 years: odds ratio [OR], 4.63; 95% confidence interval [CI], 1.22–19.99; *P* = 0.029; dyspnea: OR, 5.44; 95% CI, 1.44–23.43; *P* = 0.016).

**Table 3:** Multiple logistic regression analysis of risk factors associated with mechanical ventilator support in tetanus patients

Variable	Univariate logistic regression analysis		Multivariate logistic regression analysis	
	Odds ratio (95% confidence interval)	*P* value	Odds ratio (95% confidence interval)	*P* value
Female	0.96 (0.28–3.43)	0.949	–	–
Age > 65 years	5.28 (1.53–20.62)	0.011	4.63 (1.22–19.99)	0.029
Dyspnea	6.13 (1.76–24.33)	0.006	5.44 (1.44–23.43)	0.016

### Economic burden and medical costs

Table 4 shows the economic burden according to the following details: The median total expenditure for the patients with tetanus was US $18,011 (range, US $2680–$34,497). In particular, the expenditure for the procedure and operation, including MV management, was the most expensive (median, US $5502), followed by the expenditures for drugs and injections (median, US $3861) and diagnostic blood tests (median, US $3764).

**Table 4:** Medical costs for the 49 inpatients with tetanus

Factors	Hospitalization costs (USD)
**Medical costs for treatment, median [IQR]**	
Total cost	18,011 [2680–34,497]
Cost for patient care	136 [57–247]
Cost for medications and injections	3861 [603–7114]
Cost for procedures and operations	5502 [112–8489]
Cost for diagnostic blood tests	3764 [448–5212]
Cost for radiological tests	799 [322–1190]

*Abbreviations*: *IQR* interquartile range, *USD* United States dollars

### Clinical outcomes and features by age group

Table 5 shows the clinical outcomes and features of the older adult patients. Thirty patients were aged > 60 years, and all of the deceased were in older adult group. The need for MV was significantly greater for those aged ≥60 years than for those aged < 60 years (24 cases [80.0%] vs. 8 cases [42.1%]; *P* = 0.016). The patients aged > 60 years had longer hospitalizations than those aged < 60 years (median stay, 40.5 days [IQR, 22.0–51.0 days] vs. 21.0 days [IQR, 7.0–46.5 days]; *P* = 0.109).

**Table 5:** Clinical outcomes of the patients with tetanus by age

Outcome	Age < 60 years (*n* = 19)	Age ≥ 60 years (*n* = 30)	*P* value
Death, n (%)	0	5 (16.7%)	
Need for mechanical ventilation, *n* (%)	8 (42.1%)	24 (80.0%)	0.016
Intensive care unit stay, median [IQR] (days)	3.0 [0.0;35.0]	30.0 [9.0;39.0]	0.117
Duration of mechanical ventilation, median [IQR] (days)	0.0 [0.0;31.0]	28.5 [7.0;38.0]	0.080
Hospital stay, days [IQR] (days)	21.0 [7.0;46.5]	40.5 [22.0;51.0]	0.109
Total medical costs, median [IQR] (USD)	1634.8 [530.2;9712.6]	4906.5 [1690.1;9100.1]	0.303

*Abbreviations*: *N* number, *IQR* interquartile range, *USD* United States dollars

## Discussion

Although the prevalence of tetanus has decreased with the introduction of tetanus vaccination, tetanus still causes approximately 60,000 deaths per year worldwide [5]. In this study, we reviewed the cases of 49 patients with tetanus in two tertiary referral centers. The median age of the 49 patients with tetanus was 67.0 years; of the patients, five died and 65.3% required MV. The median hospital stay of the patients with tetanus was 39.0 days. The older adult patients required more MV, longer-term hospital care, and higher treatment cost than the young patients.

This study is comparable with previous studies in terms of the patients’ clinical characteristics. Most of the patients with tetanus were older adults, a similar finding to previous reports in developed countries [1, 6–8]. The female predominance among the patients with tetanus was consistent with the results from France and Italy, possibly due to the essential immunizations administered to men during their compulsory military service [6, 9, 10]. In our study, the most common entry site was unknown, which was comparable with other reports [11, 12]. However, other studies have reported that 90% of patients were aware of the entry site [8, 13, 14]. It is possible that the wound was too small for the patients to discover or was chronic and therefore overlooked given that an acute traumatic wound was the primary focus.

Five of our patients died during the study, resulting in a 10.2% mortality rate, which was comparable with the 13.2 and 16.5% in studies in the United States and Italy, respectively [1, 6]. A previous study in France showed a relatively high mortality rate of up to 44.5% due to the inclusion of patients with severe tetanus who were admitted to the ICU [15]. In the present study, the clinical outcomes were consistent with those of a previously published national report in Japan [7]. Nakajima et al. showed that 53.5% of all patients required MV and that most deaths occurred in the older adult group, which could be explained by the similarity in the healthcare insurance system and medical accessibility between the two countries [16]. Studies have shown that tetanus severity is associated with older adult patients, even for different healthcare insurance systems. In the United States, age > 65 years was associated with a higher risk of fatal tetanus (OR, 9.6; 95% CI, 3.6–25) [1]. In a French study of older adult ICU patients with tetanus (median age, 80 years), only age was related to the 1-year mortality rate (mean age of the deceased vs. mean age of the survivors at 1 year: 83 years [range, 81–85 years] vs. 79 years [range, 73–84 years]; *P* = 0.03) [17]. As in previous reports, our results showed higher MV and mortality rates in the older adult group. This study of tetanus patients demonstrates that older age and dyspnea may be the cause of the tendency toward MV support in the multivariate logistic regression analysis, but we did not find any studies explaining the risk factor related to MV support in patients with tetanus. We thought that further research would need to define the risk factors of mortality through a multivariate adjusted method.

Our study showed relatively lower healthcare costs for tetanus than those reported in previous studies [18–20]. However, considering that the healthcare expenditures have been relatively lower in South Korea than in other countries, our results were consistent with previous reports in that the individual economic burden from tetanus was great. Previous data suggested that the annual expenditures in 2018 for inpatient curative and rehabilitative care were US $582.4 per person in South Korea, US $1210.1 in Japan, and US $1752.4 in the United States [21]. In our study, the amount of US $18,011 per inpatient with tetanus exceeded by threefold the annual health expenditure per person in South Korea. When compared with the medical expenditures per person for other diseases in South Korea (e.g., US $3966.3 for sepsis, US $5230.8 for cardiac disease, and US $7352.5 for stomach cancer, as indicated by the Health Insurance Review and Assessment Service), the direct medical expenditures for inpatients with tetanus were significantly more expensive [22]. This does not even take into account the indirect costs of the disease, such as lost wages and productivity.

To our knowledge, no studies have reported on the Tdap or Td vaccination rate in adults in South Korea. A previous report indicated that the estimated DTaP coverage among children aged < 7 years with five vaccinations was 56.6%, despite that the Korean National Immunization Program has financed the DTaP immunization, [23]. Other reports have shown that the proportion of antitetanus immunoglobulin-G titers ≥0.1 IU/mL steadily decreased in the older adult group and that the tetanus seroprevalence rate was 19.3% for the age group > 60 years in Korea [9, 24]. The US National Center for Health Statistics in 2015 released a report that the tetanus vaccination rate for adults aged > 65 years was 56.9% over the past 10 years in the United States [25]. Choi et al. reported that all groups aged > 40 years responded with > 0.1-U/mL tetanus antibody levels after the third vaccine dose [26]. Our study showed a low vaccination rate even in patients who had experienced tetanus. Considering that the cost of three doses of the Td vaccine in South Korea is US $72 and the extraordinarily high medical cost of treating tetanus of US $18,010.5, a massive financial incentive is allotted to ensuring sufficient Td vaccination, thereby preventing tetanus and its consequent medical costs. We believe that further studies regarding the status of Tdap or Td vaccination in adults will be helpful to highlight the advantages of vaccination.

This study has a number of limitations. First, it was conducted at two local tertiary referral hospitals. Given that approximately 20 tetanus cases are reported annually in South Korea by the KCDC, the data in this study should be interpreted with caution because the cases represented approximately 20% of all tetanus cases in South Korea. Nevertheless, we believe that our data are noteworthy because the two tertiary university hospitals are national hospitals in Gyeongsangbuk-do, Jeollabuk-do, and a representative province in South Korea. Collecting data from a large number of cases would be helpful to further define the clinical manifestations and risk factors of mortality through a multivariate adjusted method. Second, the quality of the data depends on the historical data available in this retrospective cohort study. Third, we did not include healthcare costs and additional indirect costs, such as private nursing care and lost earnings during hospitalization. Nevertheless, our study is one of the few studies that have investigated the costs of treatment and economic burden caused by tetanus, and provides noteworthy information given the rarity of tetanus, which, to our knowledge, has not been investigated in a tertiary university hospital in the past 10 years.

## Conclusion

In summary, tetanus remains a severe but preventable acute infection disease, and its treatment entails high medical costs, which could be challenging for many individuals. The study observed a high rate of MV and high medical costs due to the intensive care required for older adult patients; however, tetanus immunization in adults is often overlooked. Therefore, strategies to improve tetanus vaccination should be implemented, especially for older adults.

## Data Availability

The datasets used during the present study are available from the corresponding author on reasonable request.

## Abbreviations

USD: United States dollars
Tdap: Toxoids and diphtheria and acellular pertussis vaccine
Td: Tetanus toxoids vaccine
SD: Standard deviation
IQR: Interquartile range
ICU: Intensive care unit
MV: Mechanical ventilation
SBP: Systolic blood pressure
OR: Odds ratio
CI: Confidence interval
